# Exogenous Angiotensin-(1–7) Provides Protection Against Inflammatory Bone Resorption and Osteoclastogenesis by Inhibition of TNF-α Expression in Macrophages

**DOI:** 10.1007/s00223-024-01257-6

**Published:** 2024-07-19

**Authors:** Jiayi Ren, Hideki Kitaura, Takahiro Noguchi, Fumitoshi Ohori, Aseel Marahleh, Jinghan Ma, Kayoko Kanou, Ziqiu Fan, Itaru Mizoguchi

**Affiliations:** 1 Department of Orthodontics and Dentofacial Orthopedics, Tohoku University Graduate School of Dentistry, Aoba-Ku, Sendai, Miyagi 980-8575 Japan; 2 Frontier Research Institute for Interdisciplinary Sciences, Tohoku University, Sendai, Japan

**Keywords:** Angiotensin-(1–7), Osteoclastogenesis, Peritoneal macrophage, Inflammatory mediator, MAPK pathway

## Abstract

Renin–angiotensin–aldosterone system plays a crucial role in the regulation of blood pressure and fluid homeostasis. It is reported to be involved in mediating osteoclastogenesis and bone loss in diseases of inflammatory bone resorption such as osteoporosis. Angiotensin-(1–7), a product of Angiotensin I and II (Ang I, II), is cleaved by Angiotensin-converting enzyme 2 and then binds to Mas receptor to counteract inflammatory effects produced by Ang II. However, the mechanism by which Ang-(1–7) reduces bone resorption remains unclear. Therefore, we aim to elucidate the effects of Ang-(1–7) on lipopolysaccharide (LPS)-induced osteoclastogenesis. *In vivo*, mice were supracalvarial injected with Ang-(1–7) or LPS ± Ang-(1–7) subcutaneously. Bone resorption and osteoclast formation were compared using micro-computed tomography, tartrate-resistant acid phosphatase (TRAP) stain, and real-time PCR. We found that Ang-(1–7) attenuated tumor necrosis factor (TNF)-α, TRAP, and Cathepsin K expression from calvaria and decreased osteoclast number along with bone resorption at the suture mesenchyme. *In vitro*, RANKL/TNF-α ± Ang-(1–7) was added to cultures of bone marrow-derived macrophages (BMMs) and osteoclast formation was measured via TRAP staining. The effect of Ang-(1–7) on LPS-induced osteoblasts RANKL expression and peritoneal macrophages TNF-α expression was also investigated. The effect of Ang-(1–7) on the MAPK and NF-κB pathway was studied by Western blotting. As a result, Ang-(1–7) reduced LPS-stimulated macrophages TNF-α expression and inhibited the MAPK and NF-κB pathway activation. However, Ang-(1–7) did not affect osteoclastogenesis induced by RANKL/TNF-α nor reduce osteoblasts RANKL expression *in vitro*. In conclusion, Ang-(1–7) alleviated LPS-induced osteoclastogenesis and bone resorption *in vivo* via inhibiting TNF-α expression in macrophages.

**Supplementary Information:**

The online version contains supplementary material available at 10.1007/s00223-024-01257-6.

## Introduction

The renin–angiotensin–aldosterone system (RAAS) is a critical hormonal system that is involved in the pathogenesis of conditions such as hypertension, diabetic kidney disease, and lung injury [1–3]. RAAS is mainly composed of renin, Angiotensin (Ang) I, Ang II, other downstream peptide hormones, and angiotensin-converting enzymes (ACE). Renin converts angiotensinogen into Ang I, which is subsequently transformed into the biologically active octapeptide known as Ang II by ACE. As a crucial factor in hypertension and a prime target for therapeutic interventions, Ang II induces vasoconstriction through Ang II type 1 receptors (AT1R), which is counteracted by AT2R [4]. More recently, further elements of the RAAS have been identified, with Ang-(1–7) emerging as one of the most extensively studied among them [5, 6]. Ang-(1–7) is a heptapeptide produced through the cleavage of Ang II by ACE2. It binds to its unique Mas receptor (MasR), counteracting the effects triggered by Ang II, including vasoconstriction, inflammation, and insulin resistance [7–11].

The ACE 2/Ang-(1–7)/MasR axis is tightly connected to the inhibition of bone-destructive diseases. In periodontitis, ACE2 and MasR were found to be expressed in osteoblasts and osteoclasts and treatment with Ang-(1–7) resulted in suppressed osteoclast differentiation [12]. Similarly, Ang-(1–7) improved osteoporotic changes in ovariectomized rats [13]. Furthermore, report indicates that glucocorticoid-induced osteonecrosis was alleviated through the activation of the ACE2/Ang-(1–7)/MasR axis [14]. Given that inflammation disrupts bone homeostasis and leads to bone resorption [15], the protective effects of Ang-(1–7) on bone might be attributed to its anti-inflammatory effects [16].

Osteoclasts are the cells responsible for bone resorption and osteoclastogenesis is the process through which bone marrow cells differentiate into osteoclasts after being exposed to macrophage colony-stimulating factor (M-CSF) and receptor activator of nuclear factor-kappa B ligand (RANKL) [17, 18]. M-CSF and RANKL are produced by various cell types, including osteoblasts [19]. M-CSF binds to CSF-1 receptor on macrophages that initiate subsequent intercellular communication leading to osteoclast differentiation [20]. RANKL binds to its receptor (RANK) on the surface of osteoclast precursors, driving their differentiation into mature osteoclasts. OPG is a soluble decoy receptor for RANKL and prevents its interaction with RANKL reducing osteoclast formation [21]. Tumor necrosis factor (TNF)-α promotes osteoclastogenesis by inducing the expression of M-CSF and RANKL, while also independently promoting osteoclast maturation in vitro [22, 23] and in vivo [24, 25]. Lipopolysaccharide (LPS) triggers the release of TNF-α by macrophages leading to osteoclast formation [26].

Ang-(1–7) demonstrates anti-inflammatory properties by suppressing the expression of inflammatory cytokines, especially TNF-α. In vivo*,* administration of Ang-(1–7) protected against bone resorption and reduced bone-resorptive marker (CTX) [13]. However, the effects of Ang-(1–7) on the crosstalk between osteoclasts and osteoclastogenesis promoting cells such as osteoblasts and macrophages during inflammation remain unclear. To elucidate the relationship between Ang-(1–7), inflammation, and osteoclastogenesis, we sought to explore the mechanism by which Ang-(1–7) influences LPS-induced osteoclastogenesis and demonstrated how the ACE2/Ang-(1–7)/MasR axis functions in bone resorption-related conditions.

## Materials and Methods

### Animals and Reagents

8–10-week-old male and pregnant C57BL/6 J mice were purchased from CLEA Japan (Tokyo, Japan). Male mice were randomly assigned into four groups for subcutaneous supracalvarial reagents injection, and they were the source of bone marrow-derived macrophages (BMMs) and peritoneal macrophages. Newborn mice aged 5–9 days provided primary osteoblasts from calvariae. All animal caring and experimental procedures complied with the Regulations for Animal Experiments of Tohoku University. (Certification of conformity: No. 2018DnA-049-12). LPS (Sigma-Aldrich, St. Louis, MO, USA), recombinant murine TNF-α (R&D system, Minneapolis, MN), RANKL (PEPROTECH, Cranbury, NJ), and Angiotensin-(1–7) trifluoroacetate salt (Bachem, Bubendorf, Switzerland) were used in this study. Ang-(1–7) powder was dissolved in distilled water at a stocking concentration of 100 mg/mL. Recombined mouse M-CSF was provided by an mM-CSF-producing cell line as described previously [27].

### Histological Analysis

Mice were divided into four injection groups: 1 × phosphate-buffered saline (PBS), LPS (100 μg/day), LPS + Ang-(1–7) , and Ang-(1–7) (100 μg/day) only. Each group contains at least 4 samples. The mice received a subcutaneous supracalvarial injection according to the assigned group for five consecutive days. On the sixth day, mice were sacrificed by cervical dislocation after anesthetized by isoflurane inhalation (Pfizer, New York, NY). The calvariae were dissected and fixed in 10% formalin for 3 days at 4 ℃. Micro-computed tomography (Micro-CT) scanning using ScanXmate (Comscan, Kanagawa, Japan) and 3-dimensional reconstruction for comparing the destructed areas by TRI/3D-Bon (RATOC System Engineering, Tokyo, Japan) had been conducted after fixation. Photoshop (Adobe, San Jose, CA) is used for specifying the absorption area in the calvariae images, while ImageJ (NIH, Bethesda, MD) is used to calculate the proportion of this area in a specified area. Then decalcification in 14% ethylenediaminetetraacetic acid (EDTA) was processed at room temperature for 1 week. The sagittal sutures of calvariae were divided into three equal parts from the coronal plane and subjected to a dehydration in a tissue processer (TP1020, Leica, Wetzlar, Germany). Dehydrated bone pieces were embedded in paraffin and cut into 5-µm sections perpendicular to the sagittal suture with a microtome (Leica). Sections were stained with tartrate-resistant acid phosphatase (TRAP) solution. To make the TRAP solution: Fast Red Violet, Naphthol AS-MX phosphate and N, N′-Dimethylformamide were incubated in a mixture of 0.5 M sodium tartrate dihydrate and 0.1 M sodium acetate (pH 5.0). The nucleus was counterstained with hematoxylin. All observation and picturing were performed with a digital camera DP-72-SET-B (OLYMPUS, Tokyo, Japan) and cellSens Standard (OLYMPUS). TRAP-positive multinucleated cells (with ≥ three nuclei) closely adjacent to the red-stained bone wall in the sagittal suture mesenchyme were counted manually (Supplementary Fig. 1). Statistical analysis was based on an average of 10 sections from one sample [28].

### Preparation of Primary Osteoclast and Osteoclastogenesis

Bone marrow was flushed from femora and tibiae with pre-cooled sterilized 1 × PBS by centrifuge (3000 rpm, 3 min, 4 ℃). Cells were filtered by a 40-μm cell strainer (Falcon: Corning, Corning, NY) in minimum essential medium α(α-MEM) supplied with 10% fetal bovine serum (FBS), 100U/mL penicillin, and 100 μg/mL streptomycin (Wako, Osaka, Japan), cultured with 100 ng/mL M-CSF using a petri dish (Asnol: AS ONE, Osaka, Japan) in 37 ℃ for 3 days. On the fourth day, adhesive cells were harvested as BMMs using 0.05% trypsin-EDTA (Gibco: Thermo Fisher Scientific, Rockford, IL). BMMs were seeded in a 96-well plate at a concentration of 5 × 10^4^ cells/200μL/well, and reagents were added as follows: 100 ng/mL M-CSF (negative control), M-CSF + RANKL or TNF-α (positive control), the second group plus 10 μg/mL Ang-(1–7) (about 10^−5^ M, experimental group), and M-CSF + Ang-(1–7), *n* = 4. The medium and reagents were changed and added every 2 days and cultured for 5 days in total. Cells were fixed in 10% formalin for 15 min and permeabilized with 0.1% Triton X-100 for 10 min. After staining with TRAP solution, TRAP-positive cells were observed under a light microscope and counted manually [29].

### Preparation of Primary Osteoblasts and Osteoblasts Culture

5–9-day-old neonatal mice were euthanized by inhalation of overdose isoflurane and sterilized in 70% ethanol. The calvariae were dissected between anterior and posterior fontanelles then digested and fractionated to bone chips using a collagenase isolation buffer containing 70 mM NaCl, 10 mM NaHCO_3_, 60 mM sorbitol, 3 mM K_2_HPO_4_, 1 mM CaCl_2_, 0.1% (w/v) BSA, 0.5% (w/v) glucose, and 25 mM HEPES and collagenase powder. Fractionation was carried out in a BioShaker (TAITEC, Saitama, Japan) (300 rpm 20 min, 37 ℃) using 0.2% (w/v) collagenase solution and 5 mM EDTA in 1 × PBS with 0.1% BSA. The sequence of digestion and fractionation is as follows: collagenase (Fraction 1), EDTA (Fraction 2), and collagenase (Fractions 3–4) [30]. Cells harvested from fractions 2–4, which are rich in primary osteoblasts, were seeded in a treated dish (Corning). After culture in α-MEM in 37 ℃ for 3 days, cells were harvested as mature osteoblasts and treated with LPS ± Ang-(1–7) to investigate the RANKL expression. 3-h and 48-h cultures were conducted to study the effects of short-and long-term stimulation, respectively.

### Isolation and Culture of Peritoneal Macrophages

Mice were euthanized and the peritoneum was exposed without breaking the serous membrane. A 21G needle and syringes were used to inject 6 mL ice-cold sterilized 1 × PBS beneath the peritoneal viscera over the inner side of the femur. Generally, 5 mL peritoneal lavage fluid was collected. Cells were harvested by centrifuging (1500 rpm, 8 min, 4 ℃) in 1 × PBS. Filtered cells were seeded in 12-well plates, and non-adhesive cells were washed two hours later. Then adherent cells were incubated in 37 ℃ overnight and cultured with LPS ± Ang-(1–7) for 3 days to study the effect of Ang-(1–7) on macrophages TNF-α expression.

### RNA Extraction and Real-Time Reverse Transcription-Polymerase Chain Reaction

Calvariae tissues were homogenized by stirring with ceramic beads in TRIzol reagent (Ambion: Thermo Fisher Scientific) using Micro-Smash MS-100R (TOMY SEIKO, Tokyo, Japan). Chloroform (Wako, Osaka, Japan) was added to the mixture at one-fifth volume of the TRIzol and centrifuged (14,000 rpm, 10 min, 4 ℃) to collect the supernatant containing RNA. RNA was then extracted following manufacturer’s instruction using RNeasy Kits (QIAGEN, Hilden, Germany). The cells were lysed with a 100:1 mixture of RLT and 2-mercaptoethanol and then transferred to the QIAShredder for homogenization followed by centrifugation (14000 rpm, 2 min, 4 ℃). Total RNA was dissolved in RNase-free water and stored at − 80 ℃. RNA concentration and quality was checked using a Nano-Photometer (Implen, Munich, Germany). cDNA was made by Super-script IV Reverse Transcriptase system using Oligo dT primer (Invitrogen, Thermo Fisher Scientific). Primers utilized in real-time PCR are listed in Table 1. GAPDH was selected as an internal control gene, and TB Green® Premix Ex Taq™ II was used (TaKaRa, Shiga, Japan).

**Table 1:** Primer sequences used for real-time PCR

Gene	Forward primer	Reverse primer
GAPDH	GGTGGAGCCAAAAGGGTCA	GGGGGCTAAGCAGTTGGT
TNF-α	CTGTAGCCCACGTCGTAGC	TTGAGATCCATGCCGTTG
RANKL	CCTGAGGCCCAGCCATTT	CTTGGCCCAGCCTCGAT
OPG	ATCAGAGCCTCATCACCTT	CTTAGGTCCAACTACAGAGGAAC
TRAP	AACTTGCGACCATTGTTA	GGGGACCTTTCGTTGATGT
Cathepsin K	GCAGAGGTGTGTACTATGA	GCAGGCGTTGTTCTTATT
IL-6	CTGCAAGAGACTTCCATCCAG	AGTGGTATAGACAGGTCTGTTGG

### Western Blot

Peritoneal macrophages stimulated by LPS ± Ang-(1–7) for 0, 5, 15, and 30 min were lysed in radioimmunoprecipitation assay buffer consisting of 1% protease and phosphatase inhibitor after 3 h of starvation in serum-free α-MEM. QIAShredder was used to remove cell pellets (14,000 rpm, 2 min, 4 ℃) and homogenize total protein [30]. Protein concentration was quantified with a BCA assay kit (Thermos fisher Scientific) and a microplate reader was used to measure the absorbance at 595 nm. Protein was stored in -80℃ or mixed with sample buffer and denatured at 95 ℃ for 5 min. The sample buffer was prepared by combining 9 parts of 4 × Laemmli with 1 part of 2-mercaptoethanol, and each protein sample was mixed with the sample buffer in a 3:1 volume ratio. Denatured protein samples were stored in – 20 ℃.

Protein was loaded on Precast Gels (Biorad, Hercules, CA) in 1 × Tris/Glycine/SDS buffer (120 V,1 h) and transferred to the PVDF membrane by the Trans-Blot Turbo Transfer System (Biorad). The membrane was blocked with a mixture of 4 g block ace powder and 20 mg sodium azide dissolved in 100 mL 1 × Tris -buffered saline with 1% Triton X-100 (TBS-T). Primary and secondary antibodies (Table 2) were diluted in immunoreaction enhancer solution (TOYOBO, Osaka, Japan) in specific dilutions. Membranes were washed in 1 × TBS-T for 10 min twice and 1 × TBS once prior to and after secondary antibody incubation. Membrane reacted with West Femto Maximum Sensitivity Substrate was detected by FUSION FX, captured by Evolution-capt Edge (VILBER, Collégien, France), and analyzed by ImageJ using a inserted band quantification Macro [31].

**Table 2:** Antibodies and specific dilutions used for Western blotting

Antibody products	Dilution
Beta actin monoclonal antibody	1:5000
Phospho-SAPK/JNK (Thr183/Tyr185) (98F2) Rabbit mAb	1:3000
Phospho-p44/42 MAPK (Erk1/2) (Thr202/Tyr204) Antibody	1:3000
Phospho-p38 MAPK (Thr180/Tyr182) (D3F9) XP Rabbit mAb	1:3000
Phospho-IκBα (Ser32) (14D4) Rabbit mAb #2859	1:1000
SAPK/JNK Antibody #9252	1:3000
p44/42 MAPK (Erk1/2) Antibody #9102	1:5000
p38 MAPK Antibody #9212	1:5000
IκBα Antibody #9242	1:3000
Anti-mouse antibody	1:10,000
HRP-conjugated anti-rabbit IgG antibody	1:5000–10,000

### Statistical Analysis

All data are expressed as the mean ± standard deviation. Scheffe’s test and paired *t*-test were used to assess the differences between groups. Statistical significance was defined as *p* < 0.05. Scheffe’s test is a one-way ANOVA post hoc test, and it corrects alpha for complex mean comparisons with a narrower confidential interval. Data were analyzed using the Excel statistics Statcel 3 (Microsoft, Seattle, WA).

## Results

### Ang-(1–7) Suppressed LPS-Induced Calvarial Bone Resorption and In Vivo Osteoclastogenesis

**Fig. 1:**
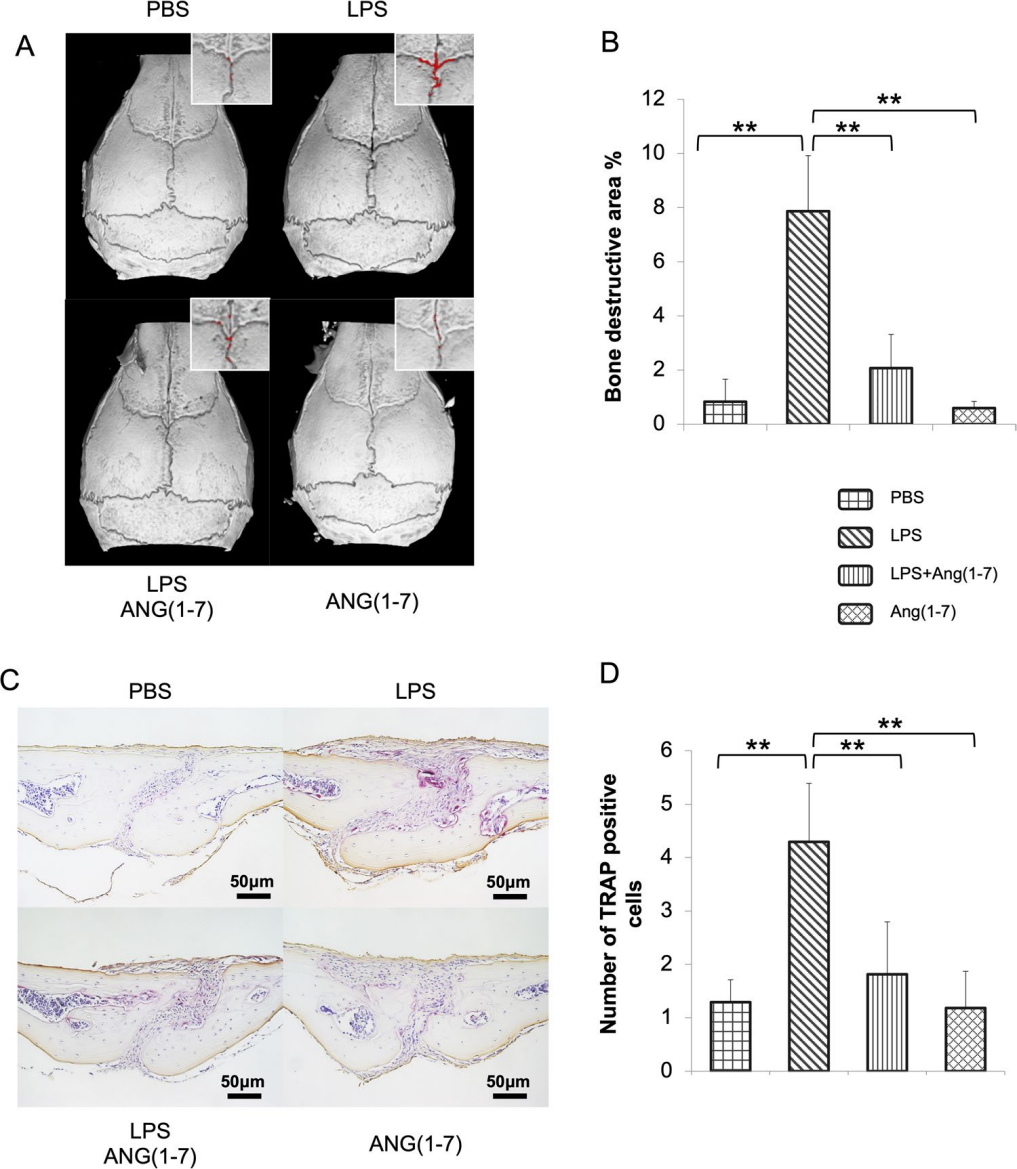
Ang-(1–7) suppressed LPS-induced calvarial bone resorption and osteoclast formation in vivo. **A** Three-dimensional reconstructed calvariae. The red area showed on the top left refers to the destructed area around the anterior-fontanelle suture, and pixels were automatically selected using Photoshop; **B** Percentage of the destructed bone area in a specific-sized square (50 × 70 pixels) was analyzed by ImageJ; **C** TRAP staining of sagittal suture mesenchyme; **D** Number of TRAP-positive cells. Data for analysis are average number of over ten valid sections from every sample. Scheffe’s test was used to determine the statistical significance of differences between groups (*n* = 4–5; **p < 0.01)

**Fig. 2:**
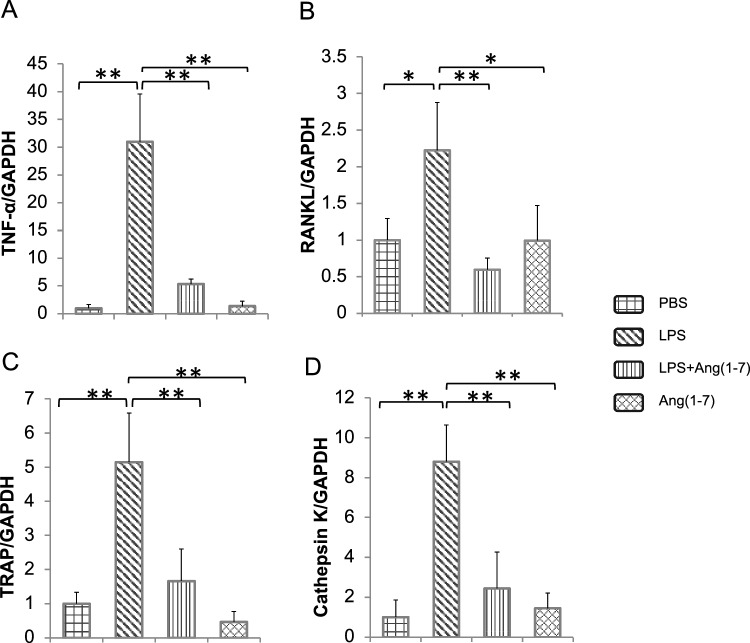
Ang-(1–7) decreased the LPS-elevated inflammatory and osteoclastic biomarkers in vivo analyzed using real-time PCR. Data are shown in the form of folds relative to the PBS group which has been standardized into 1. **A** TNF-α mRNA expression level relative to GAPDH; **B** RANKL mRNA expression level relative to GAPDH; **C** TRAP mRNA expression level relative to GAPDH; **D** CTSK mRNA expression level relative to GAPDH. Non-significant data have not been labeled. Scheffe’s test was used to determine the statistical significance of differences between groups (*n* = 4; **p < 0.01 *p < 0.05)

We dissected the injected calvariae to analyze the effect of Ang-(1–7) on bone resorption in vivo. We elevated the bone-resorbed area by Micro-CT scanning and found that LPS distinctly destructed the bone structure around the anterior suture’s junction. LPS + Ang-(1–7) reduced the extent of the destruction. Ang-(1–7) alone had no effect on the normal structure of calvaria (Fig. 1A, B). According to histological analysis of calvariae sections stained with TRAP solution, LPS stimulation increased the number of TRAP-positive multinucleated cells, while the addition of Ang-(1–7) decreased it (Fig. 1C, D). We also evaluated TNF-α, RANKL, TRAP, and Cathepsin K (CTSK) expression levels using excised calvarial tissue by real-time PCR. As a result, LPS significantly elevated the mRNA expression level of TNF-α, RANKL, TRAP, and CtsK. In comparison with this, LPS + Ang-(1–7) inhibited these inflammatory or osteoclastogenic biomarkers production responding to LPS (Fig. 2).

### Ang-(1–7) has No Effect on In Vitro Osteoclastogenesis

**Fig. 3:**
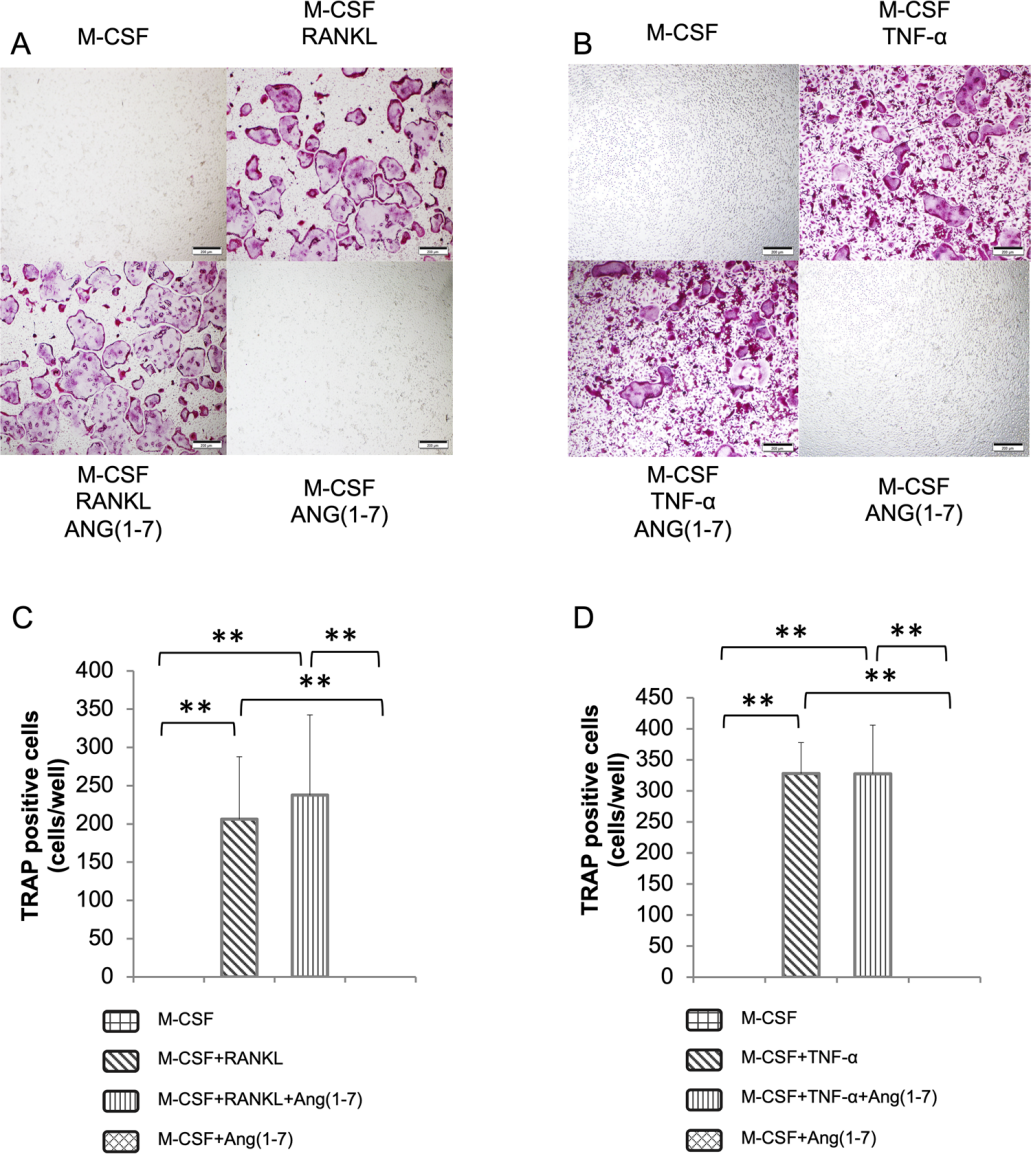
Ang-(1–7) does not affect RANKL or TNF-α-induced osteoclastogenesis. Bone marrow cells culture with M-CSF for 3 days were used as BMMs. **A** RANKL-induced osteoclastogenesis under a light scope; **B** TNF-α-induced osteoclastogenesis. **C**, **D** Manually counted number of TRAP-positive multinucleated cells; The scale bar is 200 μm. Scheffe’s test was used to determine the statistical significance of differences between groups (*n* = 4; **p < 0.01)

To investigate whether Ang-(1–7) has a direct effect on osteoclastogenesis, we added Ang-(1–7) to M-CSF + RANKL, M-CSF + TNF-α, or M-CSF alone to cultures of BMMs. Consequently, RANKL and TNF-α induced the formation of multinucleated TRAP-positive cells as expected. However, Ang-(1–7) had no significant effect on RANKL or TNF-α osteoclastogenesis as reflected by the number of TRAP-positive cells. M-CSF ± Ang-(1–7) alone did not induce any TRAP-positive cell formation. (Fig. 3).

### Ang-(1–7) Does Not Affect RANKL Expression of Osteoblasts

**Fig. 4:**
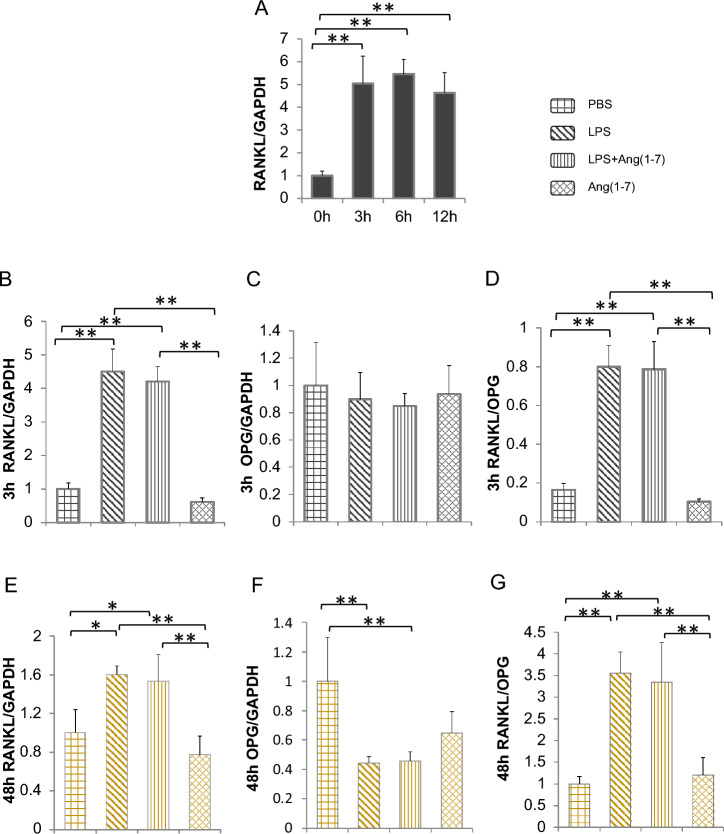
Ang-(1–7) does not affect LPS-induced RANKL expression of osteoblasts. **A** LPS-stimulated RANKL mRNA expression level relative to GAPDH in 0, 3, 6,and 12 h; **B**–**D** RANKL, OPG expression level, and RANK/OPG ratio after 3-h stimulation; **E**–**G** RANKL, OPG expression level, and RANK/OPG ratio after 48-h stimulation. Non-significant data have not been labeled. Scheffe’s test was used to determine the statistical significance of differences between groups (*n* = 4; **p < 0.01 *p < 0.05)

We cultured osteoblasts to study if Ang-(1–7) affects LPS-induced RANKL expression and osteoclast formation. Isolated primary osteoblasts were cultured with LPS for the specific period (0, 3, 6, 12 h) to evaluate the time required for LPS to induce RANKL expression. LPS induced RANKL expression after 3 h (Fig. 4A). Therefore, 3 h and 48 h were selected to investigate the effect of Ang-(1–7) on short-and long-term stimulation of LPS on RANKL production. LPS significantly upregulated RANKL expression and downregulated OPG expression, increasing the ratio of RANKL/OPG in both 3-and 48-h cultures of LPS ± Ang-(1–7) (Fig. 4B–G). However, Ang-(1–7) had no effect on LPS-induced RANKL or OPG expression.

### Ang-(1–7) Inhibits LPS-Induced TNF-α Expression, NF-κB, and MAPKs Pathway Activation in Peritoneal Macrophages

**Fig. 5:**
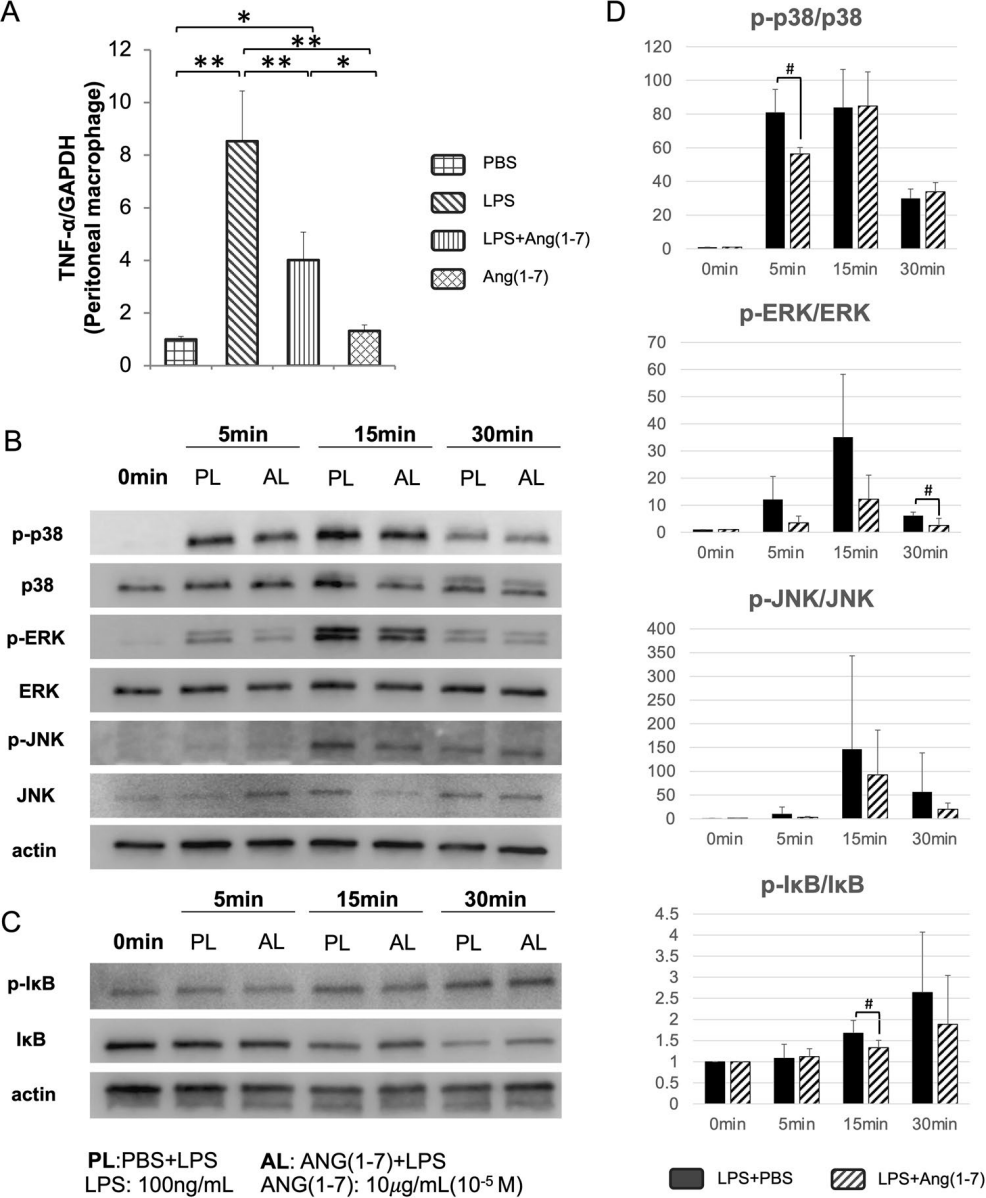
Ang-(1–7) suppressed LPS-induced TNF-α releasing and the MAPKs and NF-κB pathway activation in peritoneal macrophages. **A** Real-time PCR result of TNF-α mRNA expression level relative to GAPDH for 3 days culture (*n* = 4, *p < 0.05; **p < 0.01). **B**, **C** Western blotting for effect of Ang-(1–7) on phosphorylated proteins in relation to non-phosphorylated proteins and β-actin in the MAPKs and NF-κB pathway **D** bands quantification of each phosphorylated protein; Scheffe’s test and paired t-test were used to determine the statistical significance of differences between groups in real-time PCR and Western blotting, respectively. Non-significant data have not been labeled. (*n* = 3 for WB; #*p* < 0.05)

We measured TNF-α and IL-6 expression (Supplementary Fig. 2) from peritoneal macrophages using real-time PCR after 3 days of LPS ± Ang-(1–7) culture. Two hours after seeding, we removed the non-adhesive cells, and the adhered cells were cultured with LPS and Ang-(1–7). Consistent with our *in vivo* study, TNF-α mRNA level increased distinctly in the LPS group which was suppressed by Ang-(1–7) (Fig. 5A). Besides, we measured Ang-(1–7)’s effect on the phosphorylation of JNK, p38, and ERK1/2 from the MAPK pathway and phosphorylation of IκB from NF-κB pathway in peritoneal macrophages by Western blotting with specific antibodies. Consequently, Ang-(1–7) significantly reduced the phosphorylation of p38, ERK1/2, and IκB at different time points of stimulation but did not affect JNK (Fig. 5B–D).

## Discussion

The ACE 2/Ang-(1–7)/MasR axis has gained significant attention over the past decades due to its antagonistic role in relation to Ang II. The immunomodulatory property of RAAS emerged in the fields of oncology, transplantation, and intensive care medicine indicating its extensive interaction with inflammatory regulation [32]. Moreover, the localized expression of RAAS components in bone has been linked to various bone-destructive conditions, including but not limited to osteoporosis and periodontitis [12, 33]. Ang-(1–7) has been observed to ameliorate the deterioration of osteomicrostructure in an OVX-rat model [13]. The underlying mechanism is believed to involve anti-inflammatory and anti-osteoclastogenic properties. Given that inflammation is widely regarded as a contributor to bone degradation and that Ang-(1–7) is anti-inflammatory, our study investigated the effects of the bioactive heptapeptide Ang-(1–7) on LPS-induced inflammatory osteoclast formation and bone destruction.

Ang-(1–7) inhibits inflammation in vivo, possibly by reducing serum levels of pro-inflammatory cytokines such as TNF-α and IL-6 [34]. TNF-α is a pivotal pro-inflammatory factor released by macrophages in innate immune responses upon stimulation by LPS [35]. Given that LPS is capable of promoting inflammatory osteoclastogenesis [26], our initial focus was on investigating whether Ang-(1–7) inhibits LPS-induced bone resorption and osteoclast formation. Calvaria subcutaneously injection was chosen based on previous study and its specialty in inducing local inflammation [29]. Consequently, supracalvarial injection of LPS leads to increased osteoclast numbers and bone resorption around the suture mesenchyme which decreased in Ang-(1–7) + LPS injection compared to LPS injection alone. In vivo mRNA expression levels of TNF-α, RANKL, TRAP, and CTSK were in line with the results obtained from histological analysis, which were significantly elevated by LPS and attenuated in LPS + Ang-(1–7) group. These findings indicate that Ang-(1–7) plays a pivotal role in reducing LPS-stimulated bone resorption and osteoclast formation.

RANKL, originating from a diversity of cells including osteoblasts [19], is a potent inducer of osteoclast differentiation by binding to its receptor RANK on osteoclast precursors and pre-osteoclasts. Also, TNF-α can increase osteoclast formation as TNF receptors knockout mice experienced suppressed osteoclastogenesis reported in a previous study [36]. Its ability to induce hyperosteoclastogenesis independent of RANKL is still controversial, as Lam and colleagues stated that TNF-α cannot induce any formation of osteoclast without the permission of RANKL [37]. In this case, a micro-amount of RANKL or osteoblast/stromal cell contaminated the serum or our cultures may explain the hyperosteoclastogenic phenomenon. Considering that TNF-α and RANKL are central to osteoclastogenesis, we sought to investigate the effects of Ang-(1–7) on osteoclast formation induced by these two factors in vitro. To accomplish this, we cultured bone marrow cells with M-CSF, which serve as osteoclast precursors. The measurement result of TRAP-positive multinucleated osteoclasts suggested that Ang-(1–7) does not inhibit osteoclastogenesis by directly acting on osteoclast precursors. This finding contrasts with the previous studies [35], where 10^−7^ M (100 nM) Ang-(1–7) inhibited osteoclastogenesis. Conversely, both 10^−7^ M and 10^−5^ M (100 μM) concentrations of Ang-(1–7) exhibited no inhibitory effects in our study. Although we isolated bone marrow cells from long bones, the differences in the animal model (rat or mouse), incubation period with M-CSF (1–10 days), and M-CSF concentration (10–100 ng/mL), cell culture conditions (administration of regents such as daily treat with Ang-(1–7) or extra stimulation of LPS, and stimulation period (5–10 days)) may have gave rise to the opposing effects of Ang(1–7) in the three different studies [12, 38].

Accordingly, investigating the upstream regulatory factors becomes imperative. As described earlier, RANKL is the essential molecule facilitating communication between osteoblast and osteoclast. RANKL is tightly linked to osteoclast activation and alveolar bone loss in periodontitis [39]. A previous study demonstrated that LPS boosted RANKL expression in bone marrow stromal cells [40]. In a different context, Ang-(1–7) decreased RANKL expression in ovariectomized rats after 6 weeks of treatment [13]. Building on the evidence presented before, an intuitive experiment focusing on the effect of Ang-(1–7) on osteoblasts RANKL expression is necessary. We isolated neonatal calvarial osteoblasts and cultured them with LPS for 3, 6, or 12 h to identify the time point at which RANKL expression increases. RANKL expression increased at 3 h. Therefore, we cultured osteoblasts with LPS ± Ang-(1–7) for 3 h to assess the effect of Ang-(1–7) on LPS-stimulated RANKL expression. In addition, we conducted an identical 48-h experiment to explore long-term culture outcome. Nonetheless, Ang-(1–7) did not exhibit any effect on LPS-induced osteoblast RANKL expression in vitro.

As a pro-inflammatory cytokine substantially released from macrophage upon LPS stimulation, TNF-α is responsible for osteoclastogenesis not only through direct induction [24] but also by stimulating osteoblasts to express RANKL [41]. Ang-(1–7) has been reported to eliminate TNF-α expression from peritoneal macrophage [42]. Given that TNF-α promotes inflammatory bone resorption, we investigated Ang-(1–7)’s effect on LPS-induced TNF-α expression from peritoneal macrophages. In line with previous research and our in vivo study, Ang-(1–7) decreased TNF-α expression in peritoneal macrophages after 72 h of culture. Taking into account that TNF-α could accelerate osteoclastogenesis through both direct and indirect pathways, our study indicates that Ang-(1–7) suppresses LPS-induced osteoclast formation by inhibiting TNF-α production in macrophages.

The MAPK pathway modulates various cellular activities, including proliferation, differentiate, and survival. It is activated during inflammation in response to diverse cellular stresses [43]. The phosphorylation of key proteins ERK1/2, p38, and JNK in each pathway leads to the secretion of cytokines and chemokines in macrophages induced by LPS [44]. The NF-κB pathway is crucial to modulate inflammatory response toward various stimuli. Toll-like receptors, upon stimulation by LPS, transduce cellular signals and mediate IκBα phosphorylation downstream, thereby activating the classical NF-κB pathway. This activation prompts the cell to produce multiple factors that combat inflammation [45]. Therefore, we studied the activation of MAPK and NF-κB pathways by culturing peritoneal macrophages with LPS ± Ang-(1–7) for specific periods (0, 5, 15, 30 min) after 3 h starvation to exclude the influence of FBS. Through Western blotting analysis, all three phosphorylated proteins in MAPK pathway exhibited a rapid increase at 5 or 15 min activated by LPS, followed by a declining trend after 15 min. Among these, phosphorylation of p38 and ERK1/2 initiated by LPS was significantly reduced by Ang-(1–7), while p-JNK was not influenced by Ang-(1–7). Phosphorylated IκB showed a gentle rise until 30 min, and significant inhibition of Ang-(1–7) was observed at 15 min. The effect of Ang-(1–7) on the MAPK and NF-κB pathway activation may be responsible for reduced TNF-α expression in macrophages.

However, there may be some possible limitations in this study. To fully understand the effects of Ang-(1–7), a more systemic analysis is required, as the current animal model only involves localized inflammation. Additionally, exploring the effects of ACE2 and Mas receptor in conjunction with Ang-(1–7) would provide a more comprehensive understanding of RAAS. Addressing these limitations in future studies would result in a more accurate understanding of the effects of Ang-(1–7) and its potential as a therapeutic agent.

Ang-(1–7) is underrepresented in clinical studies despite its potential role in alleviating pathological conditions closely associated with Ang II, such as cardiovascular and renal diseases. Given its significant involvement in inflammatory mediation, particularly in inflammatory bone lesions, there is an urgent need for further investigation in the context of clinical trials targeting skeletal degenerative diseases under inflammation like periodontitis, osteoporosis, and rheumatic arthritis.

In summary, inflammation is interconnected with numerous chronic diseases, such as atherosclerosis, diabetes, and arthritis. ACE2/Ang-(1–7)/MasR axis possesses anti-inflammatory properties and holds potential benefits for bone-related conditions like osteoporosis and periodontitis. Our study has demonstrated that Ang-(1–7) is anti-inflammatory and reduces bone resorption in vivo. More importantly, we have clarified its mechanism does not involve the direct inhibition of osteoclastogenesis. Instead, Ang-(1–7) eliminates LPS-induced osteoclast formation by suppressing the pro-inflammatory cytokine TNF-α released by macrophage, and it inhibits the phosphorylation of p38, ERK1/2 in the MAPKs pathway, and phosphorylation of IκB in NF-κB pathway. Nevertheless, while promising, the development of Ang-(1–7) as a therapeutic strategy for bone-resorptive diseases necessitates further research.

## Conclusion

Collectively, in vivo, Ang-(1–7) attenuated LPS-stimulated TNF-α, RANKL, TRAP, and CTSK mRNA expressions from calvaria, and decreased osteoclast number along with bone resorption at the suture mesenchyme. In vitro, Ang-(1–7) reduced LPS-induced TNF-α expression, phosphorylation of p38 and ERK1/2 in the MAPK pathway, and phosphorylation of IκB in NF-κB pathway from peritoneal macrophages. However, Ang-(1–7) did not inhibit osteoclastogenesis directly induced by TNF-α or RANKL added to BMMs cultures. Ang-(1–7) had no effect on osteoblasts RANKL expression.

**Fig. 6:**
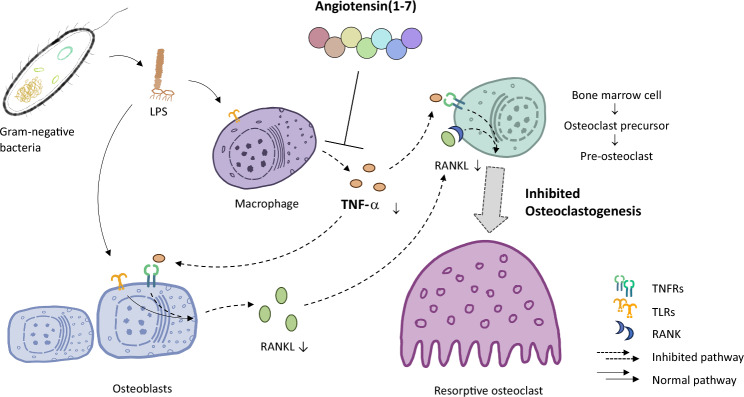
Illustration explaining that Ang-(1–7) inhibits LPS-induced osteoclastogenesis by attenuating TNF-α expression in macrophages. LPS derived from gram-negative bacteria activates macrophages to produce a substantial amount of TNF-α. Versatile TNF-α initiates osteoclast formation through two distinct pathways, (1) by binding to TNFRs on bone marrow cells, osteoclast precursors, and pre-osteoclasts, sequentially inducing osteoclastogenesis; (2) by binding to TNFRs on osteoblasts, stimulating a surge in RANKL, which is highly effective in inducing osteoclastogenesis. Ang-(1–7) exhibits an inhibitory effect on TNF-α expression in macrophages, resulting in a weaker induction of TNF-α on osteoclastogenesis

In summary, our study showed that Ang-(1–7) alleviated LPS-induced osteoclastogenesis and bone resorption via the inhibition of TNF-α expression in vivo without inhibiting osteoclastogenesis directly (Fig. 6). This process may be caused by inhibiting the intracellular activation of MAPK pathway and NF-κB pathway in macrophages leading to TNF-α expression inhibition.

## Supplementary Information

Below is the link to the electronic supplementary material.

Supplementary file1 (PDF 356 KB)

## Data Availability

Data will be made available upon reasonable request.

## Abbreviations

ACE: Angiotensin-converting enzyme
ACE2: Angiotensin-converting enzyme 2
Ang: Angiotensin
AT1R: Angiotensin II type 1 receptor
AT2R: Angiotensin II type 2 receptor
α-MEM: Minimum essential medium α
BMMs: Bone marrow-derived macrophages
BSA: Bovine serum albumin
CTSX: Cathepsin K
CTX: Carboxy-terminal cross-linked telopeptide of type 1 collagen
EDTA: Ethylenediaminetetraacetic acid
FBS: Fetal bovine serum
GAPDH: Glyceraldehyde-3-phosphate dehydrogenase
IL-6: Interleukin 6
LPS: Lipopolysaccharide
MAPK: Mitogen-activated protein kinase
MasR: Mas receptor
M-CSF: Macrophage colony-stimulating factor
Micro-CT: Micro-computed tomography
NF-kB: Nuclear Factor kappa-light-chain-enhancer of activated B cells
OPG: Osteoprotegerin
PBS: Phosphate-buffered saline
RAAS: Renin–angiotensin–aldosterone system
RANK: Receptor activator of nuclear factor-kappa B
RANKL: Receptor activator of nuclear factor-kappa B ligand
TBS: Tris-buffered saline
TBS-T: Tris-buffered saline with 1% Triton X-100
TNF-α: Tumor necrosis factor-α
TNFR: Tumor necrosis factor receptor
TRAP: Tartrate-resistant acid phosphatase
