# Supplementary material for: Exogenous Angiotensin-(1–7) Provides Protection Against Inflammatory Bone Resorption and Osteoclastogenesis by Inhibition of TNF-α Expression in Macrophages
Source: Calcif Tissue Int. 2024 Jul 19;115(4):432–44. doi: 10.1007/s00223-024-01257-6 (PMC11405502; doi:10.1007/s00223-024-01257-6)
Supplement: Supplementary file 1 — Supplementary file1 (PDF 356 KB) [file 223_2024_1257_MOESM1_ESM.pdf]

Supplement Materials

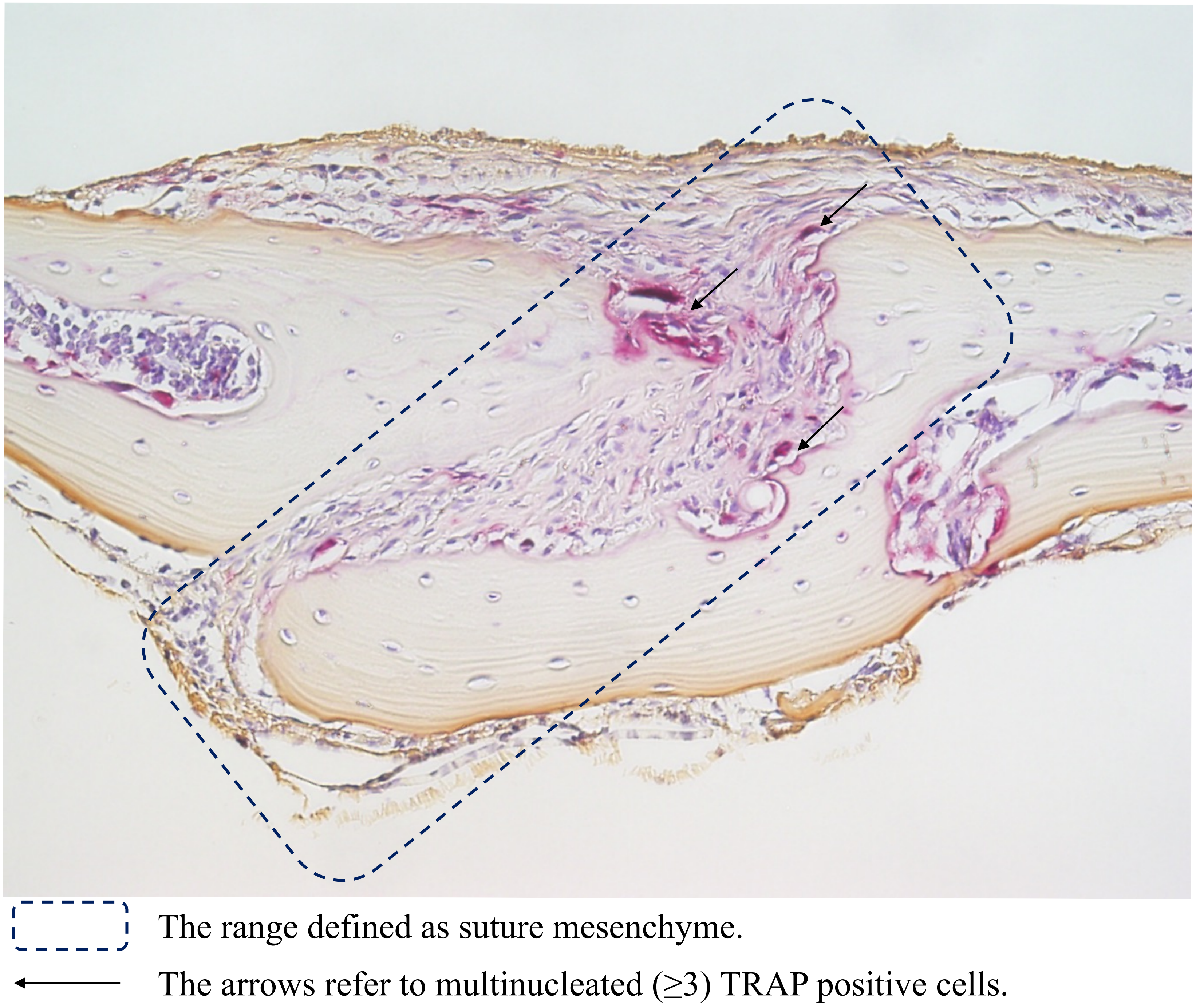

The range defined as suture mesenchyme.

The arrows refer to multinucleated (≥3) TRAP positive cells.

Supplementary Fig. 1 Method to measure TRAP-positive cells in the sagittal suture mesenchyme.

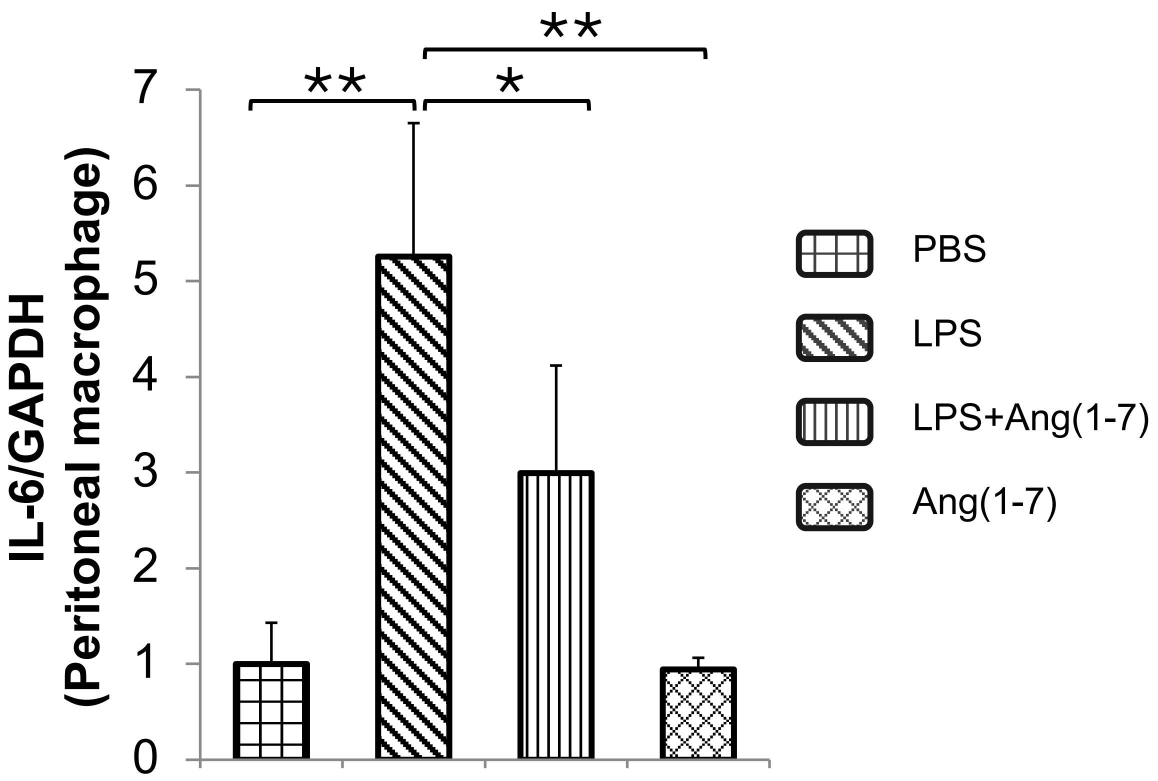

Supplementary Fig. 2 Angiotensin-(1-7)'s effect on IL-6 expression from peritoneal macrophages ($* p < 0.05$, $** p < 0.01$, $n = 4$).
